# (4-Hy­droxy-3-methyl­phen­yl)(phen­yl)methanone

**DOI:** 10.1107/S160053681302521X

**Published:** 2013-09-18

**Authors:** C. S. Dileep, V. Lakshmi Ranganatha, N. K. Lokanath, A. K. Shaukath, M. A. Sridhar

**Affiliations:** a Department of Studies in Physics, Manasagangotri, University of Mysore, Mysore 570 006, India; b Department of Chemistry, Yuvaraja’s College, University of Mysore, Mysore 570 005, India

## Abstract

In the title compound, C_14_H_12_O_2_, the benzene rings make a dihedral angle of 58.84 (12)°. In the crystal, mol­ecules are linked into chains along the *b-*axis direction by O—H⋯O hydrogen bonds. These chains are further linked by C—H⋯O hydrogen bonds, forming layers parallel to the *bc* plane.

## Related literature

For the biological activity of benzo­phenone derivatives, see: Khanum *et al.* (2004 ▶); Naveen *et al.* (2006 ▶); Selvi *et al.* (2003 ▶). For a related structure, see: Mahendra *et al.* (2005 ▶).
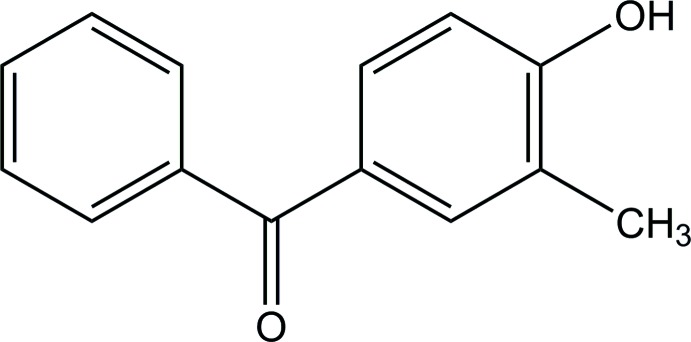


## Experimental

### 

#### Crystal data

C_14_H_12_O_2_
*M* *_r_* = 212.24
Orthorhombic, 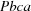

*a* = 7.7043 (4) Å
*b* = 16.3770 (8) Å
*c* = 17.7482 (9) Å
*V* = 2239.4 (2) Å^3^
*Z* = 8
Cu *K*α radiation
μ = 0.67 mm^−1^
*T* = 293 K
0.30 × 0.25 × 0.20 mm

#### Data collection

Bruker X8 Proteum diffractometer
7657 measured reflections
1828 independent reflections
1518 reflections with *I* > 2σ(*I*)
*R*_int_ = 0.037

#### Refinement

*R*[*F* ^2^ > 2σ(*F* ^2^)] = 0.046
*wR*(*F* ^2^) = 0.129
*S* = 1.03
1828 reflections
147 parameters
H-atom parameters constrained
Δρ_max_ = 0.14 e Å^−3^
Δρ_min_ = −0.15 e Å^−3^

### 

Data collection: *APEX2* (Bruker, 2006 ▶); cell refinement: *SAINT* (Bruker, 2006 ▶); data reduction: *SAINT*; program(s) used to solve structure: *SHELXS97* (Sheldrick, 2008 ▶); program(s) used to refine structure: *SHELXL97* (Sheldrick, 2008 ▶); molecular graphics: *PLATON* (Spek, 2009 ▶); software used to prepare material for publication: *SHELXL97*.

## Supplementary Material

Crystal structure: contains datablock(s) global, I. DOI: 10.1107/S160053681302521X/is5303sup1.cif

Structure factors: contains datablock(s) I. DOI: 10.1107/S160053681302521X/is5303Isup2.hkl

Supplementary material file. DOI: 10.1107/S160053681302521X/is5303Isup3.cml

Additional supplementary materials:  crystallographic information; 3D view; checkCIF report

## Figures and Tables

**Table 1:** Hydrogen-bond geometry (Å, °)

*D*—H⋯*A*	*D*—H	H⋯*A*	*D*⋯*A*	*D*—H⋯*A*
O14—H14⋯O8^i^	0.82	1.91	2.7106 (19)	166
C2—H2⋯O14^ii^	0.93	2.57	3.448 (3)	158

Symmetry codes: (i) 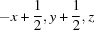; (ii) 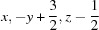.

**Fig. 1.**
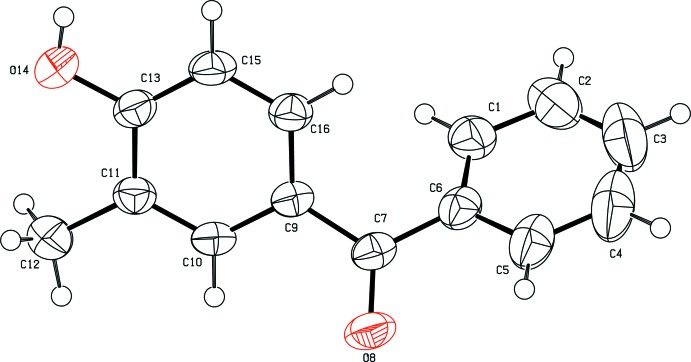
An ORTEP view of the molecule with the atom-labeling scheme. The thermal ellipsoids are drawn at the 50% probability level. H atoms are shown as small spheres of arbitrary radii.

**Fig. 2.**
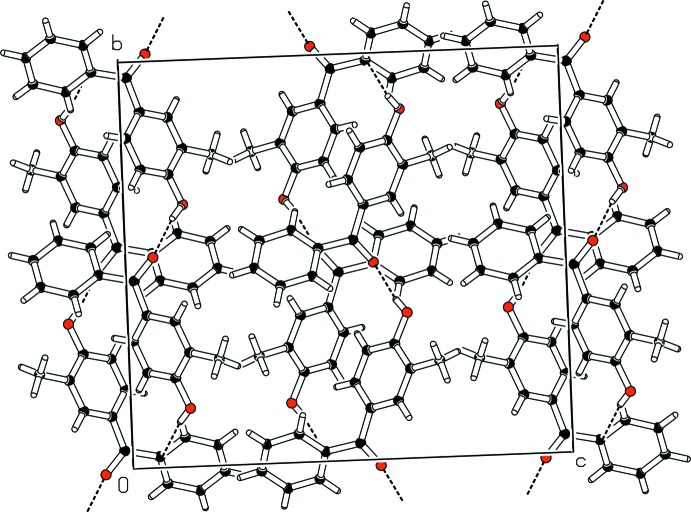
A molecular packing view of the title compound down the a-axis, showing intermolecular interactions (dashed lines).

## supplementary crystallographic information

### 1. Comment

Benzophenone and related compounds have a wide variety of biological activities such as anti-fungal and anti-inflammatory (Khanum *et al.*, 2004; Selvi *et al.*, 2003). The presence of various substituents in the benzophenone nucleus is essential to determining the quantitative structure-activity relationships of these systems. The competence of benzophenones as chemotherapeutic agents, especially as inhibitors of HIV-1 reverse transcriptase RT, cancer and inflammation, is well established and their chemistry has been studied extensively. In addition, methyl-substituted benzophenones exhibit chemotherapeutical activity against fungi. Some studies were carried out to show that methyl-substituted benzophenones exhibit anti-fungal properties (Naveen *et al.*, 2006). In view of its extensive background, the title compound was prepared and characterized by single-crystal X-ray diffraction.

In the molecular structure of the title compound (Fig. 1), bond lengths and angles do not show large deviations and are comparable with those reported for a similar structure (Mahendra *et al.*, 2005). The dihedral angle between the two benzene rings (C1–C6) and (C9–C16) is 58.84 (12)°. The crystal structure is stabilized by intermolecular C—H···O and O—H···O hydrogen bonds (Table 1 & Fig. 2).

### 2. Experimental

The title compound was synthesized by Fries rearrangement. 3-Methylphenylbenzoate was treated with anhydrous aluminium chloride (0.002 mol) as a catalyst at 150–170 °C under without solvent condition for about 2–3 h. Then the reaction mixture was cooled to room temperature and quenched with 6 N HCl in the presence of ice water. The reaction mixture was stirred for about 2–4 h, and the solid was filtered and recrystallized with acetonitrile to obtain the title compound.

### 3. Refinement

All H-atoms were located in a difference map and then were positioned geometrically (C—H = 0.93–0.96 Å and O—H = 0.82 Å). They were refined using a riding model with *U*_iso_(H) = 1.2*U*_eq_(C) or 1.5*U*_eq_(O, C_methyl_) .

### Crystal data

**Table:**

C_14_H_12_O_2_	*F*(000) = 896
*M**_r_* = 212.24	*D*_x_ = 1.259 Mg m^−^^3^
Orthorhombic, *P**b**c**a*	Cu *K*α radiation, λ = 1.54178 Å
Hall symbol: -P 2ac 2ab	Cell parameters from 1828 reflections
*a* = 7.7043 (4) Å	θ = 5.0–64.4°
*b* = 16.3770 (8) Å	µ = 0.67 mm^−^^1^
*c* = 17.7482 (9) Å	*T* = 293 K
*V* = 2239.4 (2) Å^3^	Block, colorless
*Z* = 8	0.30 × 0.25 × 0.20 mm

### Data collection

**Table:**

Bruker X8 Proteum diffractometer	1518 reflections with *I* > 2σ(*I*)
Radiation source: Bruker MicroStar microfocus rotating anode	*R*_int_ = 0.037
Helios multilayer optics monochromator	θ_max_ = 64.4°, θ_min_ = 5.0°
Detector resolution: 10.7 pixels mm^-1^	*h* = −3→8
φ and ω scans	*k* = −18→18
7657 measured reflections	*l* = −20→20
1828 independent reflections	

### Refinement

**Table:**

Refinement on *F*^2^	Secondary atom site location: difference Fourier map
Least-squares matrix: full	Hydrogen site location: inferred from neighbouring sites
*R*[*F*^2^ > 2σ(*F*^2^)] = 0.046	H-atom parameters constrained
*wR*(*F*^2^) = 0.129	*w* = 1/[σ^2^(*F*_o_^2^) + (0.0617*P*)^2^ + 0.8504*P*] where *P* = (*F*_o_^2^ + 2*F*_c_^2^)/3
*S* = 1.03	(Δ/σ)_max_ < 0.001
1828 reflections	Δρ_max_ = 0.14 e Å^−^^3^
147 parameters	Δρ_min_ = −0.15 e Å^−^^3^
0 restraints	Extinction correction: *SHELXL*, FC^*^=KFC[1+0.001XFC^2^Λ^3^/SIN(2Θ)]^-1/4^
Primary atom site location: structure-invariant direct methods	Extinction coefficient: 0.0023 (3)

### Special details

**Table:**

Geometry. Bond distances, angles *etc*. have been calculated using the rounded fractional coordinates. All su's are estimated from the variances of the (full) variance-covariance matrix. The cell e.s.d.'s are taken into account in the estimation of distances, angles and torsion angles
Refinement. Refinement on *F*^2^ for ALL reflections except those flagged by the user for potential systematic errors. Weighted *R*-factors *wR* and all goodnesses of fit *S* are based on *F*^2^, conventional *R*-factors *R* are based on *F*, with *F* set to zero for negative *F*^2^. The observed criterion of *F*^2^ > σ(*F*^2^) is used only for calculating -*R*-factor-obs *etc*. and is not relevant to the choice of reflections for refinement. *R*-factors based on *F*^2^ are statistically about twice as large as those based on *F*, and *R*-factors based on ALL data will be even larger.

### Fractional atomic coordinates and isotropic or equivalent isotropic displacement parameters (Å^2^)

**Table:**

	*x*	*y*	*z*	*U*_iso_*/*U*_eq_	
O8	0.1870 (2)	0.48369 (7)	0.06235 (8)	0.0564 (5)	
O14	0.1579 (2)	0.85740 (8)	0.13455 (8)	0.0637 (6)	
C1	0.1850 (3)	0.56879 (13)	−0.12119 (11)	0.0553 (7)	
C2	0.2200 (4)	0.54665 (17)	−0.19461 (13)	0.0756 (9)	
C3	0.3252 (4)	0.4804 (2)	−0.20900 (18)	0.0944 (11)	
C4	0.3942 (4)	0.43527 (19)	−0.15054 (18)	0.0886 (11)	
C5	0.3563 (3)	0.45538 (13)	−0.07775 (14)	0.0623 (8)	
C6	0.2522 (3)	0.52263 (11)	−0.06186 (11)	0.0457 (6)	
C7	0.2081 (2)	0.54037 (10)	0.01775 (10)	0.0407 (6)	
C9	0.1909 (2)	0.62549 (10)	0.04420 (10)	0.0374 (5)	
C10	0.0890 (2)	0.64224 (10)	0.10733 (10)	0.0385 (5)	
C11	0.0763 (2)	0.71935 (10)	0.13776 (10)	0.0412 (6)	
C12	−0.0375 (3)	0.73782 (12)	0.20447 (12)	0.0624 (8)	
C13	0.1725 (2)	0.78203 (10)	0.10381 (10)	0.0426 (6)	
C15	0.2755 (3)	0.76672 (11)	0.04110 (11)	0.0456 (6)	
C16	0.2830 (2)	0.68939 (11)	0.01104 (10)	0.0431 (6)	
H1	0.11670	0.61430	−0.11120	0.0660*	
H2	0.17280	0.57630	−0.23430	0.0910*	
H3	0.35000	0.46600	−0.25850	0.1130*	
H4	0.46650	0.39120	−0.16080	0.1060*	
H5	0.40030	0.42390	−0.03850	0.0750*	
H10	0.02750	0.59980	0.12970	0.0460*	
H12A	−0.09550	0.68880	0.22030	0.0940*	
H12B	−0.12210	0.77820	0.19070	0.0940*	
H12C	0.03250	0.75820	0.24510	0.0940*	
H14	0.21640	0.88990	0.11020	0.0950*	
H15	0.33940	0.80880	0.01940	0.0550*	
H16	0.34990	0.67960	−0.03160	0.0520*	

### Atomic displacement parameters (Å^2^)

**Table:**

	*U*^11^	*U*^22^	*U*^33^	*U*^12^	*U*^13^	*U*^23^
O8	0.0647 (10)	0.0356 (7)	0.0688 (9)	0.0039 (6)	0.0086 (7)	0.0147 (6)
O14	0.0935 (12)	0.0353 (7)	0.0622 (9)	−0.0083 (7)	0.0170 (8)	−0.0035 (6)
C1	0.0597 (13)	0.0525 (11)	0.0538 (11)	−0.0129 (10)	0.0040 (10)	0.0005 (9)
C2	0.0883 (19)	0.0862 (17)	0.0524 (12)	−0.0340 (15)	0.0057 (13)	−0.0043 (12)
C3	0.096 (2)	0.111 (2)	0.0763 (18)	−0.0373 (19)	0.0327 (17)	−0.0442 (18)
C4	0.0747 (18)	0.0860 (19)	0.105 (2)	−0.0047 (15)	0.0238 (17)	−0.0464 (18)
C5	0.0519 (13)	0.0530 (12)	0.0819 (15)	−0.0007 (10)	0.0089 (12)	−0.0169 (11)
C6	0.0397 (10)	0.0400 (10)	0.0574 (11)	−0.0072 (8)	0.0047 (9)	−0.0047 (8)
C7	0.0341 (10)	0.0342 (9)	0.0538 (10)	−0.0001 (7)	0.0016 (8)	0.0057 (8)
C9	0.0352 (9)	0.0332 (9)	0.0438 (9)	−0.0001 (7)	−0.0005 (7)	0.0061 (7)
C10	0.0381 (10)	0.0347 (9)	0.0428 (9)	−0.0014 (7)	0.0007 (8)	0.0109 (7)
C11	0.0440 (11)	0.0389 (9)	0.0407 (9)	0.0017 (7)	0.0011 (8)	0.0068 (7)
C12	0.0773 (16)	0.0518 (12)	0.0582 (12)	0.0033 (11)	0.0216 (11)	0.0039 (10)
C13	0.0496 (11)	0.0332 (9)	0.0451 (10)	0.0001 (8)	−0.0030 (8)	0.0041 (7)
C15	0.0468 (11)	0.0375 (10)	0.0526 (10)	−0.0088 (8)	0.0057 (9)	0.0097 (8)
C16	0.0419 (11)	0.0398 (9)	0.0476 (10)	−0.0025 (8)	0.0061 (8)	0.0054 (8)

### Geometric parameters (Å, º)

**Table:**

O8—C7	1.231 (2)	C11—C13	1.402 (2)
O14—C13	1.354 (2)	C13—C15	1.390 (3)
O14—H14	0.8200	C15—C16	1.375 (3)
C1—C6	1.396 (3)	C1—H1	0.9300
C1—C2	1.379 (3)	C2—H2	0.9300
C2—C3	1.378 (4)	C3—H3	0.9300
C3—C4	1.380 (4)	C4—H4	0.9300
C4—C5	1.365 (4)	C5—H5	0.9300
C5—C6	1.391 (3)	C10—H10	0.9300
C6—C7	1.482 (3)	C12—H12A	0.9600
C7—C9	1.477 (2)	C12—H12B	0.9600
C9—C16	1.395 (2)	C12—H12C	0.9600
C9—C10	1.395 (2)	C15—H15	0.9300
C10—C11	1.377 (2)	C16—H16	0.9300
C11—C12	1.504 (3)		
			
O8···C6^i^	3.385 (3)	C13···H16^vii^	2.8700
O8···C7^i^	3.383 (2)	C16···H1	2.8000
O8···C1^i^	3.169 (3)	H1···C9	2.8200
O8···O14^ii^	2.7106 (19)	H1···C16	2.8000
O14···O8^iii^	2.7106 (19)	H1···H16	2.5200
O8···H10	2.5600	H2···O14^viii^	2.5700
O8···H5	2.6200	H3···C10^ix^	3.0100
O8···H14^ii^	1.9100	H3···H10^ix^	2.4500
O14···H12B	2.7100	H5···O8	2.6200
O14···H12C	2.7200	H5···C7^v^	3.1000
O14···H2^iv^	2.5700	H10···O8	2.5600
C1···C16	3.159 (3)	H10···H12A	2.3700
C1···O8^i^	3.169 (3)	H10···H3^vi^	2.4500
C5···C7^v^	3.522 (3)	H12A···H10	2.3700
C6···O8^i^	3.385 (3)	H12B···O14	2.7100
C7···C7^i^	3.525 (2)	H12C···O14	2.7200
C7···C5^v^	3.522 (3)	H14···H15	2.2900
C7···O8^i^	3.383 (2)	H14···O8^iii^	1.9100
C16···C1	3.159 (3)	H14···C7^iii^	3.0200
C1···H16	2.7300	H15···H14	2.2900
C6···H16	2.7300	H15···C10^x^	3.0700
C7···H14^ii^	3.0200	H16···C1	2.7300
C7···H5^v^	3.1000	H16···C6	2.7300
C9···H1	2.8200	H16···H1	2.5200
C10···H3^vi^	3.0100	H16···C11^x^	3.0500
C10···H15^vii^	3.0700	H16···C13^x^	2.8700
C11···H16^vii^	3.0500		
			
C13—O14—H14	109.00	C9—C16—C15	120.39 (17)
C2—C1—C6	119.9 (2)	C2—C1—H1	120.00
C1—C2—C3	119.8 (2)	C6—C1—H1	120.00
C2—C3—C4	120.6 (3)	C1—C2—H2	120.00
C3—C4—C5	120.0 (3)	C3—C2—H2	120.00
C4—C5—C6	120.4 (2)	C2—C3—H3	120.00
C1—C6—C7	121.87 (18)	C4—C3—H3	120.00
C5—C6—C7	118.73 (18)	C3—C4—H4	120.00
C1—C6—C5	119.32 (19)	C5—C4—H4	120.00
O8—C7—C9	119.71 (16)	C4—C5—H5	120.00
C6—C7—C9	120.58 (15)	C6—C5—H5	120.00
O8—C7—C6	119.71 (15)	C9—C10—H10	119.00
C7—C9—C10	119.43 (15)	C11—C10—H10	119.00
C7—C9—C16	121.90 (15)	C11—C12—H12A	109.00
C10—C9—C16	118.53 (15)	C11—C12—H12B	110.00
C9—C10—C11	122.36 (15)	C11—C12—H12C	109.00
C10—C11—C12	122.32 (15)	H12A—C12—H12B	110.00
C12—C11—C13	119.95 (15)	H12A—C12—H12C	109.00
C10—C11—C13	117.73 (15)	H12B—C12—H12C	109.00
O14—C13—C11	116.76 (15)	C13—C15—H15	120.00
O14—C13—C15	122.31 (15)	C16—C15—H15	120.00
C11—C13—C15	120.92 (16)	C9—C16—H16	120.00
C13—C15—C16	120.05 (17)	C15—C16—H16	120.00
			
C6—C1—C2—C3	−1.8 (4)	C6—C7—C9—C16	−28.8 (2)
C2—C1—C6—C5	1.0 (3)	C7—C9—C10—C11	175.79 (15)
C2—C1—C6—C7	−175.6 (2)	C16—C9—C10—C11	0.1 (3)
C1—C2—C3—C4	0.8 (5)	C7—C9—C16—C15	−174.39 (17)
C2—C3—C4—C5	1.0 (5)	C10—C9—C16—C15	1.2 (3)
C3—C4—C5—C6	−1.8 (4)	C9—C10—C11—C12	178.23 (17)
C4—C5—C6—C1	0.8 (3)	C9—C10—C11—C13	−1.0 (2)
C4—C5—C6—C7	177.5 (2)	C10—C11—C13—O14	179.90 (15)
C1—C6—C7—O8	142.2 (2)	C10—C11—C13—C15	0.7 (3)
C1—C6—C7—C9	−38.4 (3)	C12—C11—C13—O14	0.7 (2)
C5—C6—C7—O8	−34.4 (3)	C12—C11—C13—C15	−178.57 (18)
C5—C6—C7—C9	145.05 (18)	O14—C13—C15—C16	−178.60 (17)
O8—C7—C9—C10	−24.9 (2)	C11—C13—C15—C16	0.6 (3)
O8—C7—C9—C16	150.64 (17)	C13—C15—C16—C9	−1.5 (3)
C6—C7—C9—C10	155.66 (17)		

Symmetry codes: (i) −*x*, −*y*+1, −*z*; (ii) −*x*+1/2, *y*−1/2, *z*; (iii) −*x*+1/2, *y*+1/2, *z*; (iv) *x*, −*y*+3/2, *z*+1/2; (v) −*x*+1, −*y*+1, −*z*; (vi) −*x*+1/2, −*y*+1, *z*+1/2; (vii) *x*−1/2, −*y*+3/2, −*z*; (viii) *x*, −*y*+3/2, *z*−1/2; (ix) −*x*+1/2, −*y*+1, *z*−1/2; (x) *x*+1/2, −*y*+3/2, −*z*.

### Hydrogen-bond geometry (Å, º)

**Table:**

*D*—H···*A*	*D*—H	H···*A*	*D*···*A*	*D*—H···*A*
O14—H14···O8^iii^	0.82	1.91	2.7106 (19)	166
C2—H2···O14^viii^	0.93	2.57	3.448 (3)	158

Symmetry codes: (iii) −*x*+1/2, *y*+1/2, *z*; (viii) *x*, −*y*+3/2, *z*−1/2.
